# 3,4-Dioxygenated xanthones as antifouling additives for marine coatings: in silico studies, seawater solubility, degradability, leaching, and antifouling performance

**DOI:** 10.1007/s11356-023-26899-1

**Published:** 2023-05-02

**Authors:** Cátia Vilas-Boas, Elisabete R. Silva, Diana Resende, Beatriz Pereira, Gonçalo Sousa, Madalena Pinto, Joana R. Almeida, Marta Correia-da-Silva, Emília Sousa

**Affiliations:** 1 Laboratory of Organic and Pharmaceutical Chemistry, Department of Chemical Sciences, Faculty of Pharmacy, University of Porto, Rua Jorge Viterbo Ferreira, 228, 4050-313 Porto, Portugal; 2 CIIMAR/CIMAR-Interdisciplinary Centre of Marine and Environmental Research, University of Porto, Avenida General Norton de Matos, 4450-208 Matosinhos, Portugal; 3 BioISI - Biosystems & Integrative Sciences Institute, Faculty of Sciences, University of Lisbon, Campo Grande, 1749-016 Lisbon, Portugal; 4 CERENA - Center for Natural Resources and Environment, Instituto Superior Técnico, University of Lisbon, Av. Rovisco Pais 1, 1049-001 Lisbon, Portugal

**Keywords:** Biofouling, Antifouling, Marine pollution, Bioaccumulation, Degradation, Coatings, Xanthones, Organic synthesis

## Abstract

Marine biofouling pollution is a process that impacts ecosystems and the global economy. On the other hand, traditional antifouling (AF) marine coatings release persistent and toxic biocides that accumulate in sediments and aquatic organisms. To understand the putative impact on marine ecosystems of recently described and patented AF xanthones (xanthones 1 and 2), able to inhibit mussel settlement without acting as biocides, several in silico environmental fate predictions (bioaccumulation, biodegradation, and soil absorption) were calculated in this work. Subsequently, a degradation assay using treated seawater at different temperatures and light exposures was conducted for a period of 2 months to calculate their half-life (DT_50_). Xanthone 2 was found to be non-persistent (DT_50_ < 60 days) at 50 μM, contrary to xanthone 1 (DT_50_ > 60 days). To evaluate the efficacy of both xanthones as AF agents, they were blended into four polymeric-based coating systems: polyurethane- and polydimethylsiloxane (PDMS)-based marine paints, as well as room-temperature-vulcanizing PDMS- and acrylic-based coatings. Despite their low water solubility, xanthones 1 and 2 demonstrated suitable leaching behaviors after 45 days. Overall, the generated xanthone-based coatings were able to decrease the attachment of the *Mytilus galloprovincialis* larvae after 40 h. This proof-of-concept and environmental impact evaluation will contribute to the search for truly environmental-friendly AF alternatives.

**Supplementary Information:**

The online version contains supplementary material available at 10.1007/s11356-023-26899-1.

## Introduction

Marine biofouling pollution describes the community of micro and macro-organisms that settle and grow on external submerged or semi-submerged surfaces (natural or artificial) (Dafforn et al. 2011). Despite being a natural process, marine biofouling carries a lot of disadvantages, namely (1) the speed of ships is significantly reduced due to an increase in the drag friction that can reach up to 40%; (2) additional fuel is required to overcome the resistance from the biofilm, which can represent up to 60% of the total vessel’s operational cost; (3) consumption of extra fuel releases more greenhouse gases such as CO_2_, SOx, and NOx; (4) additional expenses not only from the extra fuel but also from cleaning the hulls to remove the adhered organisms; (5) higher maintenance reduces the ship’s availability to navigate; and (6) transportation of species and pathogens to different places causing a public health problem (Flemming 2002; Silva et al. 2021).

Since marine biofouling entails huge environment, economic, and human health problems, countermeasures must be considered. In the 1960s, the organotin tributyltin (TBT), which was initially used as a co-toxicant in high-performance copper coatings, demonstrated a powerful antifoulant capacity (Dafforn et al. 2011). However, through biomagnification, all marine predators were exposed to TBT, which caused a diversity of side effects such as hormonal issues, genetic abnormalities, interference with shell growth, and reproduction of bivalves (Matthiessen 2019). In addition, TBT was implicated in the massive decline of the French and English native oyster fisheries. Due to all these adverse effects, TBT was globally banned in 2008. Subsequently, paint manufacturers have developed effective TBT-free formulations that retain copper as the main biocidal agent. Copper-based AF coatings (CuAFs) have become the most widely used materials in marine industry. These coatings exert biocidal activity by discouraging the attachment of larval forms of shell-fouling organisms with the release of Cu^2+^ ions (Omae 2003). Currently, 90% of the CuAFs in use contain at least 30% of copper, it is estimated that 420,000 tons of copper are being spent per year. Even though copper is 1000 times safer than TBT, copper can be a problem for aquatic life when present in such large quantities, affecting the development of marine invertebrate larvae or bioaccumulating in tissues of commercially valuable species. Also, booster biocides (herbicides and pesticides) began being employed as enhancers of the CuAFs (Sakkas et al. 2002). However, they were considered persistent in the aquatic environment due to their affinity to soil/sediments, and organism tissues, being toxic to the periphyton community and other marine organisms (Batista-Andrade et al. 2018; Thomas et al. 2002).

**Fig. 1:**
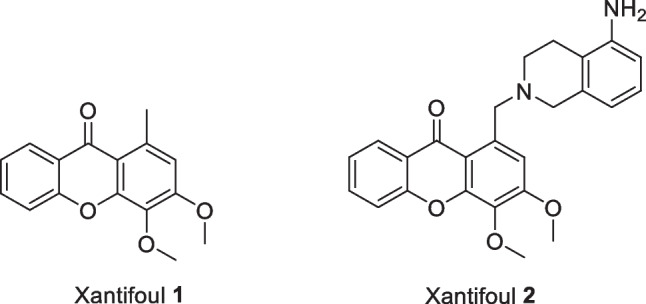
Chemical structure of antifouling synthetic xanthones 1 and 2 (Resende et al. 2021)

Xanthones have shown a variety of activities with great potential for therapeutic and commercial applications due to their versatile framework (Pinto et al. 2021). In the last decade, some natural and synthetic xanthones were described with interesting AF effects (Vilas-Boas et al. 2021; Resende et al. 2021, 2020; Almeida et al. 2020; Sun et al. 2013; Li et al. 2013; Nong et al. 2015). Particularly, synthetic xanthones 1 (Xantifoul 1) and 2 (Xantifoul 2) (Fig. 1) emerged as promising candidates (Resende et al. 2021) to replace copper due to their significant anti-settlement activity against *Mytilus galloprovincialis* larvae without causing any lethality. However, few studies can be found regarding the environmental fate of promising natural and nature-inspired AF compounds, as is the case of butenolide (Chen et al. 2015), capsaicin (Wang et al. 2014, 2020), 3,4-dihydroxyxanthone (Vilas-Boas et al. 2021), gallic acid persulfate (Vilas-Boas et al. 2020), zosteric acid (Newby et al. 2006; Vilas-Boas et al. 2017), and among others (Xu et al. 2022; Turley et al. 2000; Huang et al. 2014). However, without extensive environmental studies regarding the environmental impact on new AF compounds, these technologies will not survive the increased regulatory scrutiny and will never be marketable (Ranke and Jastorff 2000). Herein, to validate the potential of xanthones 1 and 2 as AF eco-friendly additives for commercial application, a multidimensional evaluation combining environmental fate and functionality assays were further studied.

First, a preliminary in silico environmental fate assessment was performed, followed by experimental determination of seawater solubility and degradation. Both xanthones were incorporated in marine and non-marine coatings, namely polyurethane (PU)- and polydimethylsiloxane (PDMS)-based marine paints, and room-temperature-vulcanizing silicone (RTV)-PDMS and acrylic (AV)-coatings. Leaching assays were performed for a period of 45 days and the presence of the xanthones 1 and 2 in water was quantified by high-performance liquid chromatography (HPLC). To pre-evaluate in laboratory conditions the AF effectiveness of xanthones 1 and 2 after incorporation in the generated coatings, the successful attachment of *M. galloprovincialis* larvae was assessed for 15 and 40 h.

This work takes a step forward in the development of real eco-friendly AF strategies by presenting environmental compatibility and functionality studies of two AF synthetic xanthones (water solubility, adsorption in soil, bioaccumulation and bioconcentration in organism tissues, persistence, anti-macrofouling activity, and release to artificial seawater (ASW) after their successful incorporation in several polymeric coatings), fulfilling an important gap by compiling and standardizing relevant data for ECHA approval and replacement of hazardous biocides (ECHA 2018).

## Materials and methods

### General

Xantthones 1 and 2 were obtained according to the previously described synthesis (Resende et al. 2020, 2021). Stock solutions were prepared in MeCN for Xantifoul 1 and in methanol (MeOH) with 0.1% triethylamine (TEA) for Xantifoul 2, and stored in eppendorf tubes at − 20 °C. Ultra-pure water (UPW, pH 6.1) was obtained from the Milli-Q System (Millipore). Natural seawater (TSW, pH 7.8, salinity 33.3%) was collected in the Interdisciplinary Centre of Marine and Environmental Research (CIIMAR), treated by UV light and carbon filters, and passed through a 0.45 µm syringe filter before use. Artificial seawater (ASW, pH 8.3) was prepared by diluting of sera salt (sera marine salt, German) in distilled water (33 g/L). All reagents and solvents were purchased from Sigma Aldrich, Merck, and Fluka, and had no further purification process. Acetonitrile (MeCN) for HPLC gradient grade was acquired from Biosolve BV (Valkenswaard, Netherlands).

### HPLC analysis

Analyses of solubility, degradation, and leaching samples were performed on Thermo SCIENTIFIC SpectraSYSTEM equipped with a SpectraSYSTEM UV-8000 diode-array detector (DAD), a SpectraSYSTEM P4000 pump, and a SpectraSYSTEM AS3000 autosampler, conducted on a Fortis BIO C18 column (Fortis Technologies, 5 µm, 250 × 4.6 mm), using ChromQuest 5.0™ software. The mobile phase consisted of MeCN: UPW containing 0.1% of formic acid (FA) in a proportion of 50:50 *v/v* for Xantifoul 1 (retention time 19 min) and 25:75 *v/v* for Xantifoul 2 (retention time 16 min). These mobile phases were chosen to separate each xanthone from the components of polyurethane and silicone polymeric matrix present in leaching waters at a retention time < 10 min. After mixing, mobile phases were passed through a 0.45 µm filter and degassed before use by an ultrasonic cleaner (Sonorex Digitec, Bandelin). Chromatographic conditions were set at a constant flow rate of 1 mL/min in isocratic mode, the injection volume was 20 μL, the column was maintained at room temperature (rt), and the detection wavelength was set as 239 and 242 nm for Xantifoul 1 and 2, respectively. Calibration curves for both compounds (three replicates) were prepared within the ranges of 1–200 μM and injected three times into the HPLC system. Calibration graphics were constructed by plotting the mean peak area versus concentration. The two HPLC methods for each xanthone were properly developed and validated according to the ICH Guidance for Industry Q2 (R1) (ICH 2005) through several parameters, namely specificity/selectivity, linearity, precision, accuracy, range, limits of detection (LOD), and quantification (LOQ) and used for the several assays (see supplementary material).

### Water solubility

Briefly, 1 mg of each compound was added to 500 μL of UPW and TSW in eppendorf tubes to obtain saturated solutions and was stirred for 1 h at 24 ± 1 °C, according to the shake flask method (OECD 1995). Solutions were passed through a 0.45 μm membrane filter, and the filtrated samples were evaporated to dryness under nitrogen purge by a sample concentrator Stuart® SBHCONC/1 and resuspended in 200 μL of MeCN for Xantifoul 1 and MeOH with 0.1% TEA for Xantifoul 2. Solutions were analyzed by HPLC–DAD, and the peak area of each solution was interpolated into a calibration curve. Solution concentrations were determined from the means obtained from the three repeated injections. This procedure was performed in triplicate. Solubility was defined according to United States Pharmacopeia (USP) (USP 2008-2009).

### Degradation in seawater

Degradation of xanthones 1 and 2 in treated TSW (10 mL) was evaluated in several conditions of temperature and light: 4 and 18 °C in the dark, and 25 °C in the presence and absence of light (Vilas-Boas et al. 2021). Samples were prepared to contain a maximum of 1% of organic solvent in a final concentration in TSW of 50 μM. After 2 months, each sample was extracted by solid phase extraction (SPE) using OASIS® HLB 6 cc cartridge (according to the procedure described in the “Solid-phase extraction procedure” section. Collected fractions containing chloroform were reduced to dryness under nitrogen purge in a sample concentrator and resuspended in 10 mL of MeCN for Xantifoul 1 and MeOH with 0.1% TEA for Xantifoul 2. Solutions were passed through a polytetrafluoroethylene (PTFE) filter and injected into the HPLC–DAD, and the peak area of each solution was interpolated into a calibration curve. Solution concentrations were determined from the means obtained from the three repeated injections of each sample. The procedure was made in triplicate.

### In silico environmental fate predictions

Quantitative structure–activity relationship models prediction for biodegradability was calculated with the Biodegradation Probability Program BIOWIN™ v4.10, an individual model of the Estimation Programs Interface (EPI) Suite™ (developed by U.S. Environmental Protection Agency and Syracuse Research Corporation). The sediment–water partition coefficient (Log K_oc_) was calculated through KOCWIN™ v2.00, using the molecular connectivity index, a more robust method. The octanol–water partition coefficient (Log K_ow_) was calculated through KOWWIN™ v1.68 to evaluate bioaccumulative potential. The BCFBAF™ v3.01 program was used to estimate the ratio of compounds’ concentration in fish tissues, through its concentration in water (wet weight) and to predict its bioconcentration factor (BCF).

### Preparation of xanthone-based coatings

Xanthones were immobilized in two representative biocide-free commercial marine paints for proof-of-concept, namely a foul-release polydimethylsiloxane (PDMS) HEMPASIL X3+ 87500 (base resin 87509/curing agent 98950) and a polyurethane (PU)-based paint (base resin F0032/curing agent 95580), both consisting of two-component systems and generously provided by Hempel, A/S (Copenhagen, Denmark). In addition, xanthones were also incorporated in two purchased non-marine coating systems, room-temperature-vulcanizing polydimethylsiloxane (RTV)-PDMS (RTV11, MOMENTIVE, Waterford, NY, USA), and an acrylic (AV) (VERKODUR, Ref. 690195, KORELAX, Trofa, Portugal). These non-marine coating systems were selected as model systems to better assess the compatibility with coating systems, to reveal any masked effect from the intrinsic properties of the marine coatings, and to evaluate the influence of the presence of xanthones in the coatings’ anti-settlement properties.

All xanthone-based coating systems were prepared in accordance with a previous developed direct incorporation (DI) methodology (Vilas-Boas et al. 2021). In brief, Xantifoul 1 was prior dissolved in dichloromethane (99.9%, Honeywell) to provide solutions with compound contents of 9.31, 12.58, 6.81, and 10.56 wt.%, which were further added and blended into the PU, PDMS, RTV-PDMS, and AV coating components, respectively, and in the exact amounts to yield the desired Xantifoul 1 contents in those wet formulation systems. Similarly, a previous dissolution in methyl pyrrolidone (99.5%, Acros Organics) was performed for Xantifoul 2, resulting in solutions with Xantifoul 2 contents of 9.35, 9.24, and 9.91 wt.%, which were further added and blended into the PDMS, RTV-PDMS, and AV coating components, respectively (c.f. Table 2). The proportions of volume of the paint components base/curing agent used were 2/1 and 17.8/2.2 for the PU and PDMS marine paints, and 199/1 and 3/1 for the RTV-PDMS and AV wet systems, respectively, in accordance with the instructions provided by the coating components suppliers. The xanthone-based coating formulations were optimized to avoid compromising the original appearance of the commercial coating films, such as apparent gloss and adhesion on the substrates used in this study, namely coated polyvinyl chloride (PVC) plates for the 45-day leaching tests and coated 24-well microplates for anti-macrofouling activity evaluation.

### Leaching assays

Immersions of PVC-coated plates (3.5 × 6 cm) with the developed PU, PDMS, RTV-PDMS, and AV coating systems in 0.5 L of ASW (pH 8 ± 0.3) were performed under continuous stirring (100 rpm) for a minimum period of 45 days at room temperature ranged between 18 and 21 °C, using a previously optimized stirring method (Ferreira et al. 2020). The obtained leaching waters were stored in the refrigerator (4 °C) until further analysis. For each coating formulation, the stirring test was performed in duplicate independent tests. Leaching waters were extracted by SPE using OASIS® HLB 6 cc cartridge according to the procedure described in the “Solid-phase extraction procedure” section. Chloroform solutions were dried under nitrogen purge in a sample concentrator and resuspended in 300 μL of MeCN for Xantifoul 1 and MeOH with 0.1% TEA for Xantifoul 2. Solutions were passed through a PTFE filter and injected into the RP-HPLC–DAD, and the peak area of each solution was interpolated into a calibration curve. Solution concentrations were determined from the means obtained from the three repeated injections of each sample.

### Solid-phase extraction procedure

Water samples from leaching and abiotic degradation assays containing xanthones 1 and 2 were extracted by SPE cartridges according to the following procedure: conditioning with 6 mL of MeOH; equilibration of the cartridge with 6 mL of water; elution of the sample through the column; finally, extraction of the compounds retained in the cartridge with 10 mL of chloroform and collected for a glass vial. The chloroform solutions were reduced to dryness under nitrogen purge by a sample concentrator and resuspended in their respective solvent and adequate volume. A recovery higher than 95% for this extractive procedure was obtained for a known concentration (50 µM in TSW) (Figs. S1 and S2).

### *Mytilus galloprovincialis *larvae anti-settlement bioassays

Competent *M. galloprovincialis* plantigrades with exploring behavior, previously collected in Memory beach (N41°13′51.5′′, W8°43′15.5′′) at low tide, were selected in the laboratory and transferred to the previously coated 24-well microplates with the generated PDMS, RTV-PDMS, and AV coatings containing xanthones 1 and 2, where the wells were filled with TSW. Each coating was tested in four replicates (wells) with five plantigrades per well. A negative control (AF agent-free coating system) was included. After 15 and 40 h, the percentage of larval settlement was determined by the presence/absence of efficiently attached byssal threads, produced by each individual in each coating condition.

### Statistical analysis

All data were first checked for normality (Kolmogorov–Smirnov test) and homogeneity of variances (Levene’s test), and appropriated transformations were made when necessary. Data from the abiotic degradation and anti-settlement screenings were analyzed using a one-way analysis of variance (ANOVA) followed by a Dunnett test against the control (*p* < 0.05). The software IBM SPSS Statistics 21 was used for statistical analysis.

## Results and discussion

### In silico environmental fate predictions

In silico environmental fate parameters (biodegradation, sediment–water partition, and bioaccumulation) of xanthones 1 and 2 were obtained through EPI Suite™, one of the most used free software in the registration process for biocides (Table 1).

**Table 1:** Calculated biodegradability, sediment–water partition (Log Koc), bioaccumulation (Log Kow), and bioconcentration factor (BCF) of xanthones 1 and 2 by EPI Suite™

Compound	Ultimate biodegradation (3)^1^	Aerobic conditions (4)^1^	Anaerobic conditions (7)^1,2,3^	KOCWIN™ (Log Koc)	KOWWIN™ (Log Kow)	BCFBAF™ (BCF) (L/kg wet-wt)
Xantifoul 1	Weeks–months (2.33)	Days–weeks (3.60)	NRB (− 0.02)	3.15	4.04	55.51
Xantifoul 2	Recalcitrant (1.61)	Weeks (2.99)	NRB (− 1.55)	4.82	4.24	76.28

^1^BIOWIN™ criteria: predicted time for ultimate biodegradation (3) and primary biodegradation in aerobic conditions (4) and predicted probability for fast biodegradation in anaerobic conditions (7); for (3) and (4), values > 5 = hours, > 4 = days, > 2 = months, < 2 = recalcitrant. For (7), values < 0.5 = not readily biodegradable; ^2^*RB*, readily biodegradable; ^3^*NRB*, not readily biodegradable

Under these BIOWIN™ criteria, xanthones 1 and 2 are flagged as not readily biodegradable in aerobic and anaerobic conditions, taking several weeks to be degraded, in agreement with our experimentally obtained hydrolytic results (“Water degradation” section).

According to KOCWIN™, KOWWIN™, and BCF criteria, Xantifoul 1 was predicted to have moderate sorption to soil/sediment, due to its Log Koc values between 2.5 and 3.4. In contrast, Xantifoul 2 was predicted to be tightly bound to soil/sediments (Log Koc values higher than 3.5). The obtained Log Kow value (higher than 3.5) indicates some potential affinity for lipidic matrices and a tendency to bioaccumulate. Low BCF values (lower than 2000 L/kg) were obtained for both xanthones, reinforcing their reduced bioconcentration potential in marine organisms’ tissues (Vilas-Boas et al. 2021).

### Solubility in water

Water solubility assessment is of extreme value during the development of AF agents since compounds with high water solubility are expected to be less adsorbed into sediments and bioaccumulate in fatty tissues. On the contrary, low water solubility allows a tight bond to the hydrophobic matrices of marine coating, providing a slow release to the aquatic environment (Vilas-Boas et al. 2021). For that reason, the water solubility of xanthones 1 and 2 in UPW and TSW at 24 ± 1 °C was evaluated (Table 2).

**Table 2:** Water solubility of xanthones 1 and 2 in UPW and TSW at 24 ± 1 °C quantified by HPLC

Compound	Matrix	Result (mg/L)^1^
Xantifoul 1	UPW	0.06 ± 0.01
	TSW	0.07 ± 0.02
Xantifoul 2	UPW	4.00 ± 0.02
	TSW	1.40 ± 0.03

*UPW*, ultra-pure water (pH 6.1); *TSW*, treated seawater (pH 7.8, salinity 33.3%). ^1^Mean values ± SD of three independent experiences

Xanthones 1 and 2 were classified as “Practically insoluble” according to USP solubility criteria in both waters. However, the water solubility of Xantifoul 2 is about 60 and 20 times higher than Xantifoul 1 in UPW and TSW, respectively. This can be explained due to the amine group. The water solubility of Xantifoul 2 is also almost three times higher in UPW than TSW, a phenomenon that could be explained by the lower pH of UPW, protonating the amine, and by the absence of salts on this aqueous matrix.

### Water degradation

**Fig. 2:**
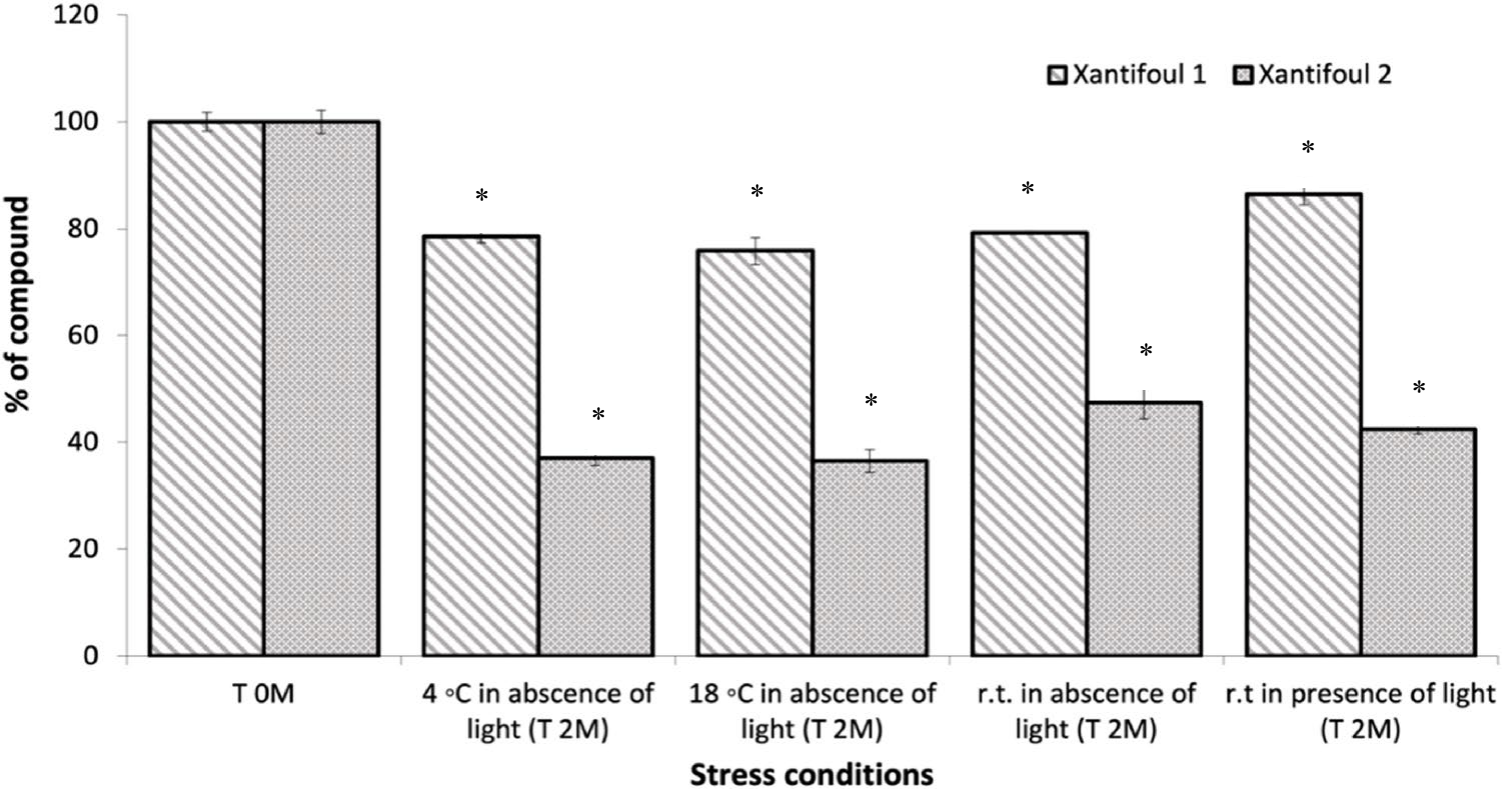
Degradation (%) of xanthones 1 and 2 (50 μM) in treated seawater (TSW) quantified by HPLC (mean values ± SD of three independent experiences) after 2 months (T 2 M) exposure to several stress conditions. *Indicates significant difference (SD) at *p* < 0.05 (Dunnett test) against the negative control (T 0 M). rt, room temperature; T 0 M, initial time; T 2 M, two months

To infer the non-persistence potential of xanthones 1 and 2 in aquatic environments, both xanthones (50 μM) were exposed to several stress conditions of temperature and light (4 and 18 °C in the dark, and 25 °C in the presence and absence of light) for 2 months (T 2 M) to mimic a natural degradative process and to obtain their DT_50_ in TSW (Fig. 2).

Xantifoul 1 was shown to be persistent with a DT_50_ > 60 days in treated TSW, similar to the emergent biocide Econea® when exposed to the same stress conditions (Vilas-Boas et al. 2021). In contrast, more than 50% of Xantifoul 2 was degraded after T 2 M in all the conditions. Overall, Xantifoul 2 possesses a DT_50_ in TSW of 48.7 days (4 and 18 °C) to 57.8 days (rt) in absence of light, and 54.8 days (rt) in presence of light, being classified as non-persistent (DT_50_ < 60 days in treated TSW). However, the formation of Xantifoul 2 transformation products were not detected by HPLC–DAD using 50 μM as the initial concentration.

### Xanthone-based coatings

The objective of developing xanthone-based coatings was to demonstrate their compatibility with commercial marine and non-marine coating systems, PU, PDMS-based marine paints, and RTV-PDMS and AV coatings, respectively, as well as to determine the limiting xanthones contents supported by each system to ensure the preservation of the original properties of the coatings, thus acting as AF additives. Several coating formulations were iteratively optimized, focusing on solvent compatibility and xanthone content to reach this objective. Table 3 shows the optimized coating formulation systems obtained and used for further proof-of-concept. Furthermore, due to Xantifoul 2’s high incompatibility with the PU-based system, no PU-based coating formulation could be prepared for this compound. It exhibited a high reactivity with the PU components system, which was attributed to the intrinsic isocyanate-based coating components’ reactivity with the primary amine functional group present in Xantifoul 2. This incompatibility, primarily manifested by a faster curing rate and agglomeration, disrupts the original properties of the paint system, entailing a thorough reformulation of the original system to produce a completely different paint system. This reformulation was not possible due to the supplier’s confidentiality requirements, which made the total composition of the paint components unknown.

**Table 3:** Xanthone-based coating formulations

Coating formulation	Base/curing agent ratio (*v*/*v*)	AF agent content (wt.%)^*^
Xantifoul 1-PU	2/1	2.0 ± 0.01
Xantifoul 1-PDMS	17.8/2.2	0.52 ± 0.02
Xantifoul 1-RTV-PDMS	199/1	0.53 ± 0.02
Xantifoul 1-AV	3/1	0.55 ± 0.02
Xantifoul 2-PDMS	17.8/2.2	0.53 ± 0.02
Xantifoul 2-RTV-PDMS	199/1	0.55 ± 0.02
Xantifoul 2-AV	3/1	1.0 ± 0.02

*AF*, antifouling; *AV*, acrylic; *PDMS*, polydimethylsiloxane; *PU*, polyurethane; *RTV-PDMS*, room-temperature-vulcanizing-polydimethylsiloxane; wt.%, percent by mass; *xanthone content in the wet coating formulation

### Leaching assays

**Fig. 3:**
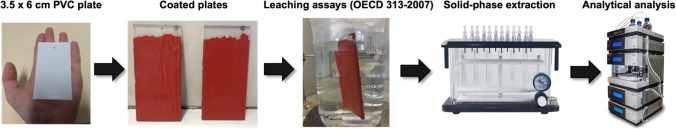
Leaching study of marine polymeric coatings containing xanthones

After the successful incorporation of both xanthones in marine polymeric coatings (PU and PDMS), leaching assays were performed and their release to ASW was analyzed by RP-HPLC–DAD, after SPE (Fig. 3).

The amount of xanthones detected on the water is given in Table 4.

**Table 4:** Leaching results of xanthones 1 and 2 direct incorporated in several polymeric coatings, after a period of 45 days in contact with ASW (0.5 L) quantified by HPLC

Coating formulation	AF agent amount in PVC plates (mg)^1^	Amount of AF agent detected in water (mg)^1^	Content of detected AF agent in water (wt.%)^1^
Xantifoul 1-PU	29.05 ± 0.64	0.49 ± 0.04	1.67 ± 0.16
Xantifoul 1-PDMS	6.10 ± 0.57	1.03 ± 0.36	16.66 ± 3.12
Xantifoul 2-PDMS	6.00 ± 0.15	1.47 ± 0.11	24.79 ± 2.44

*AF*, antifouling; *ASW*, artificial seawater; *UP*W, ultra-pure water; *wt.%*, percent by mass: (amount of detected agent in water/amount of AF agent in plates) x 100; *PDMS*, polydimethylsiloxanes; *PU*, polyurethane; *PVC*, polyvinyl chloride. ^1^Mean values ± standard deviation (SD) of two independent experiences

As expected from the assessed water solubility, Xantifoul 1 presented a leaching value lower than 2%, indicating the potential to generate long-lasting PU-based coatings. On the other hand, a premature leaching value of ~ 17 and ~ 25% from PDMS-based marine coatings of xanthones 1 and 2, respectively, indicating possible short time effects in this type of marine coating, also suggests that other immobilization strategies would be required to provide long-term effects (Vilas-Boas et al. 2021).

#### Anti-macrofouling effectiveness of developed marine coating formulations

**Fig. 4:**
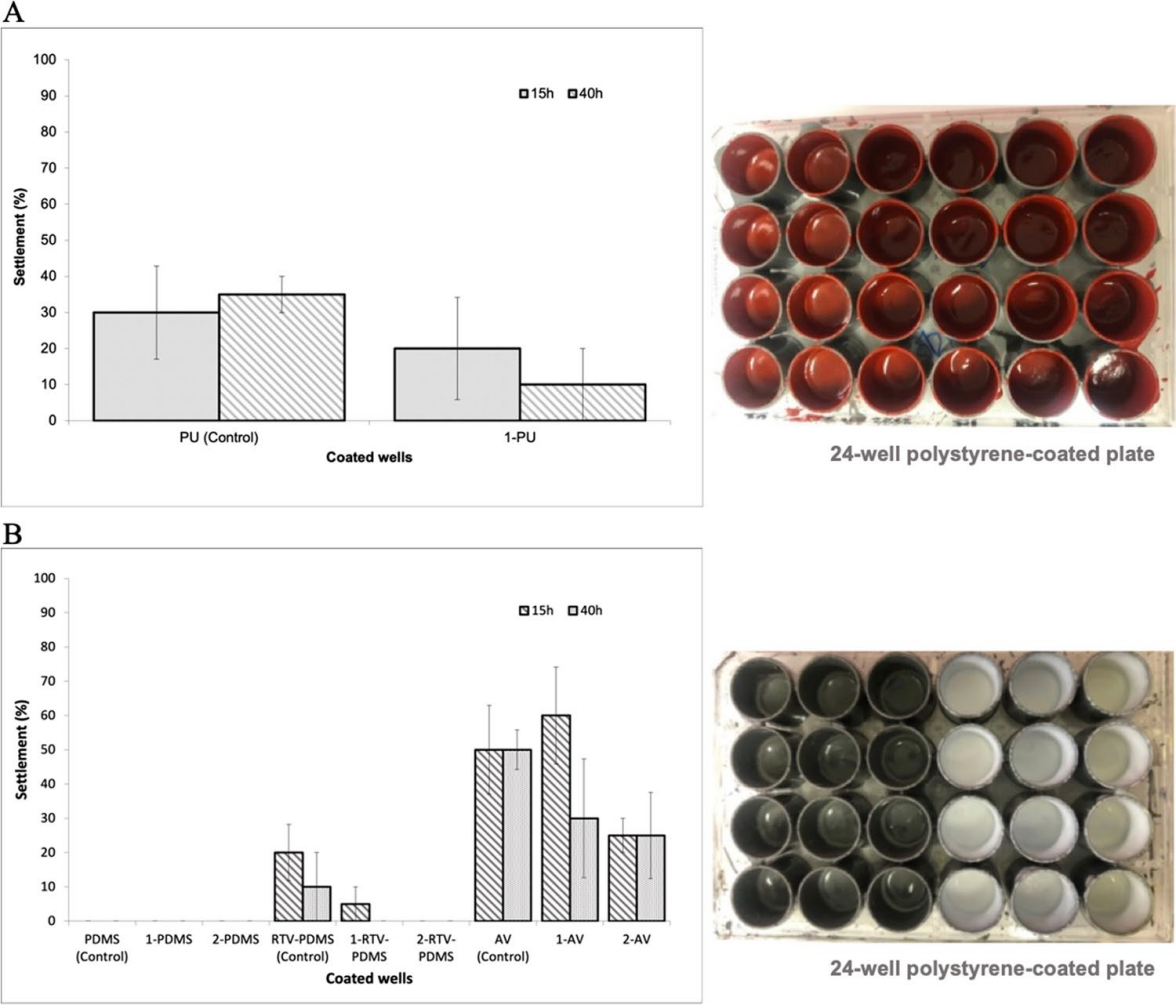
Percentage of settlement after 15 and 40 h of *Mytilus galloprovincialis* larvae (mean values of four replicates with standard error of mean (SEM) in the presence of **A** polyurethane (PU, red coating)-based coating containing Xantifoul 1 (1.8 wt.%); and **B** polydimethylsiloxane (PDMS, grey coating), room-temperature polydimethylsiloxane (RTV-PDMS, grey coating), and acrylic (AV, white coating)-based coatings containing Xantifoul 1 or Xantifoul 2 (0.5 wt.%). *Indicates significant difference at *p* < 0.05 (Dunnett test) against control

The mussel *M. galloprovincialis* is a highly invasive and global major fouling organism, due to its quick spread and its ability to displace and outcompete native mussels, causing negative economic impacts for marine industries, including shipping (Neves et al. 2020; Carl et al. 2011). For that reason, the settlement inhibition of *M. galloprovincialis* larvae on the several generated coatings formulations was also evaluated for 15 and 40 h (Fig. 4A and B) to understand the antifouling effectiveness of these new AF compounds after being incorporated into coatings.

PU marine coating containing Xantifoul 1 (Fig. 4A) seems to be effective against the settlement of mussel larvae, inhibiting larval settlement after 40 h (10% larval settlement), in contrast to the control (compound-free formulation), which showed 35% larval settlement.

Regarding PDMS-based AF marine coating formulations (Fig. 4B), a high anti-settlement effect was observed in the wells coated with the compound-free formulations, providing non-informative results regarding the AF effect of xanthones 1 and 2 after incorporation. The same results caused by this non-stick coating were also observed in previous anti-settlement assays with a nature-inspired persulfate AF compound (Vilas-Boas et al. 2020). Thus, complementary assays were performed with other optimized coating formulations such as RTV-PDMS- and AV-based to evaluate the influence of xanthones in the coatings’ anti-settlement properties, as well as to overcome any masked effect from the intrinsic properties of the marine paints (c.f. Table 3). It was possible to observe significant differences in the larval settlement on the wells coated with xanthones 1- and 2-based-RTV-PDMS coating compared to the compound-free RTV-PDMS coatings (Fig. 4A). In fact, 100% inhibition of *M. galloprovincialis* larvae settlement was observed on both RTV-PDMS-based coatings containing xanthones 1 and 2, after 40 h, in contrast to the compound-free RTV-PDMS coatings which exhibited 10% of larvae settlement. For the AV-based coating, 30% and 20% larvae settlement were observed for xanthones 1 and 2, respectively, in contrast to the compound-free AV-based coating which exhibited 50% of larvae settlement. These results demonstrate the remaining ability of both compounds in reducing the mussel larval settlement efficiently in laboratory conditions after being incorporated in different coating formulations and matrices. The potential of xanthones 1 and 2 was confirmed to be used as additives for biofouling protection and generate safer, environment-friendly, practical, and low-cost AF coatings.

## Conclusions

Marine coatings present a risk to leach out directly to the aquatic environment the AF agents added to their formulations. After leaching into ASW, a build-up of persistent and toxic compounds may occur in large marinas with a low water exchange, posing a significant risk to the organisms (Koning et al. 2020). The environmental fate and ecotoxic impacts of AF agents depend essentially on their high affinity for soil/sediments, organisms’ tissues, and degradation, a reflection of their physicochemical characteristics and the surrounding environment.

Xanthones 1 and 2 presented negligible solubility in water (< 100 mg/L). AF agents with low water solubility have the advantage of tightly binding to marine coatings, providing a slow release to marine environments. However, low water solubility may be associated with a tendency to adsorb into sediments and particles suspended in water (high Log Koc), and a tendency to bioaccumulate in fatty tissues (high Log Kow) (Qian et al. 2015). Most biocides as Chlorothalonil, Dichlofluanid, Econea®, Irgarol 1051®, and Sea-Nine 211® have Log Kow and Log Koc values higher than 3.5 and 4.5, respectively, which is the reason why tend to have more affinity into the soil and biological membranes. Most of them also present reported toxicity against several marine species, boosted by their persistence and accumulation in aquatic environments (Amara et al. 2018). In in silico environmental assays, both xanthones were predicted to have moderate affinity for fats/lipids (Log Kow > 3), Xantifoul 1 was flagged with a moderate tendency to be adsorbed into soil/sediments (Log Koc 2.5—3.4), and Xantifoul 2 was predicted to tightly bound to soil/sediments (Log Koc > than 3.5). In the future, in vitro assays regarding bioaccumulation and soil adsorption should be considered. In vitro bioaccumulation tests may be performed by HPLC analysis (OECD Test No. 117), as applied for the booster biocide Irgarol 1051® (Lam et al. 2006), or by using aquatic organisms such as *Mytilus galloprovincialis*, *Crangon crangon*, and *Tetraselmis suecica*, accordingly to assays performed with the biocides Econea®, Selektope®, and Irgarol 1051® (Dyer et al. 2006; Hilvarsson et al. 2009; Oliveira et al. 2016). Soil adsorption studies can be evaluated according to the OECD Test No. 106, a complex methodology using a batch equilibrium method with different soil types, already applied on the biocides Chlorothalonil, Dichlofluanid, Diuron, and Irgarol 1051® (Lam et al. 2006; Viana et al. 2019; Voulvoulis et al. 2002).

Both xanthones were also predicted to be not readily biodegradable in aerobic and anaerobic conditions in silico. However, in vitro hydrolysis studies, conducted in this work in treated TSW, demonstrate that Xantifoul 2 is non-persistent, with a DT_50_ < 60 days, contrary to the in silico predictions and to Xantifoul 1. These results highlight Xantifoul 2 as a better environmental alternative compared to Xantifoul 1 when it comes to developing less persistent (DT_50_ < 60 days) AF agents. These results illustrate the importance of degradation studies during the development of new antifoulants since in-silico results, although extremely useful at an early stage, are limited to specific conditions and environments inputs.

The potential of two nature-inspired AF xanthones (Xantifoul 1 and Xantifoul 2) to be incorporated as AF additives in several polymeric coating systems was proven, with suitable leaching behaviors after 45 days. Generated polyurethane-based coating containing Xantifoul 1 presented the lowest leaching of Xantifoul 1 to water (< 2%), reinforcing its potential as a long-lasting additive. All the generated coatings allowed the maintenance of xanthones bioactivity against the settlement of the mussel *M. galloprovinciallis* larvae. Both compounds exhibited similar anti-settlement activity. Overall, this study allows us to take a step forward towards the development of eco-friendly AF coatings and decrease the existing gap of new nature-inspired compounds without scrutinized environmental impacts, thus preventing the introduction of new potent but also harmful AF agents in the market to replace copper and booster biocides.

## Supplementary Information

Below is the link to the electronic supplementary material.

Supplementary file1 (DOCX 126 KB)

## Data Availability

Not applicable.
