# Supplementary material for: 3,4-Dioxygenated xanthones as antifouling additives for marine coatings: in silico studies, seawater solubility, degradability, leaching, and antifouling performance
Source: Environ Sci Pollut Res Int. 2023 May 2;30(26):68987–97. doi: 10.1007/s11356-023-26899-1 (PMC10212850; doi:10.1007/s11356-023-26899-1)
Supplement: Supplementary file 1 — Supplementary file1 (DOCX 126 KB) [file 11356_2023_26899_MOESM1_ESM.docx]

Supplementary Material

3,4-dioxygenated xanthones as antifouling additives for marine coatings: in silico studies, seawater solubility, degradability, leaching, and antifouling performance

Cátia Vilas-Boas^1,2^, Elisabete R. Silva^3,4^, Diana Resende^1,2^, Beatriz Pereira^3^, Gonçalo Sousa^1^, Madalena Pinto^1,2^, Joana R. Almeida^2^, Marta Correia-da-Silva^1,2*^, Emília Sousa^1,2^

^1^Laboratory of Organic and Pharmaceutical Chemistry, Department of Chemical Sciences, Faculty of Pharmacy, University of Porto, Rua Jorge Viterbo Ferreira, 228, 4050-313 Porto, Portugal; [up201507429@edu.ff.up.pt](mailto:up201507429@edu.ff.up.pt) (C.V.-B.), [dresende@ff.up.pt](mailto:dresende@ff.up.pt) (D.R.), [up201504718@ff.up.pt](mailto:up201504718@ff.up.pt) (G.S.), [madalena@ff.up.pt](mailto:madalena@ff.up.pt) (M.P.), [esousa@ff.up.pt](mailto:esousa@ff.up.pt) (E.S.), [m_correiadasilva@ff.up.pt](mailto:m_correiadasilva@ff.up.pt) (M.C.-d.-S.)

^2^CIIMAR/CIMAR-Interdisciplinary Centre of Marine and Environmental Research, University of Porto, Avenida General Norton de Matos, 4450-208 Matosinhos, Portugal; [jalmeida@ciimar.up.pt](mailto:jalmeida@ciimar.up.pt) (J.R.A.)

^3^BioISI - Biosystems & Integrative Sciences Institute, Faculty of Sciences, University of Lisbon, Campo Grande, 1749-016 Lisboa, Portugal; [ersilva@fc.ul.pt](mailto:ersilva@fc.ul.pt) (E.S.R.), [beatrizmmgpereira@gmail.com](mailto:beatrizmmgpereira@gmail.com) (B.P.)

^4^CERENA - Center for Natural Resources and Environment, Instituto Superior Técnico, University of Lisbon, Av. Rovisco Pais 1, 1049-001 Lisboa, Portugal

* Correspondence: [m_correiadasilva@ff.up.pt](mailto:m_correiadasilva@ff.up.pt) (M.C.-d.-S.)

**Index**

1. HPLC Method validation…………………………………………..…........………………………….………... 3

2. Recovery of the extractive procedure.…………....………………………...……………………………..…... 4

3. References…………………...………………………………………………………………………...…………..5

**HPLC method validation**

The new analytical method for xanthones **1** and **2** dissolved in methanol (MeOH) and MeOH with 0.1% triethylamine (TEA), respectively, was validated for parameters such as linearity, range, accuracy, and precision, according to the ICH Guidance for Industry Q2(R1) [1], allowing to quantify xanthones after degradation and leaching assays. The developed method was demonstrated to be linear (R^2^ >0.98) and sensitive (Table **S1**).

**Table S1.** Linear regression and sensitivity data of the newly developed method.

| **Compound** | **Range (µM) ^1^** | **Linear Regression** | **R^2^** | **LOD (µM)** | **LOQ (µM)** |
| --- | --- | --- | --- | --- | --- |
| Xantifoul **1** | 1 - 200 | y = 58136x − 49489 | 0.999 | 0.3 | 1 |
| Xantifoul **2** |  | y = 106992x - 495293 | 0.992 | 0.3 | 1 |

LOD: limit of detection; LOQ: limit of quantification; ^1^Analyses were carried out in triplicate.

The accuracy and precision were obtained from the analysis of the quality control solutions (10, 50, 150 µM), with acceptable accuracies (between 80 and 120%) and relative standard deviation (RSD) values for the intra-day and inter‐day precision lower than 20% (Table **S2**).

**Table S2.** Accuracy and intra- and inter-day variability (precision) of the newly developed method.

| **Compound** | **Concentration (µM) ^1^** | **Accuracy**  **(% ± SD)** | **Intra-Day Variability (RSD ± SD)** | **Inter-Day Variability (RSD ± SD)** |
| --- | --- | --- | --- | --- |
| Xantifoul **1** | 10 | 98.7 ± 2.8 | 6.1 ± 3.8 | 15.6 ± 3.3 |
|  | 50 | 100.9 ± 11.5 | 15.0 ± 1.3 | 14.4 ± 5.6 |
|  | 150 | 101.7 ± 4.5 | 3.0 ± 1.2 | 4.8 ± 1.3 |
| Xantifoul **2** | 10 | 116.3 ± 3.2 | 4.1 ± 2.2 | 6.1 ± 1.6 |
|  | 50 | 92.5 ± 6.0 | 3.2 ± 1.4 | 7.8 ± 0.1 |
|  | 150 | 82.0 ± 7.0 | 6.6 ± 1.8 | 9.3 ± 2.1 |

TSW: natural seawater; SD: standard deviation; RSD: relative standard deviation; UPW: ultrapure water. ^1^Mean values ± standard deviation of three independent experiences.

Overall, the developed methods were found to be precise, accurate, highly sensitive, and linear in the established range, allowing a suitable detection and quantification of xanthones 1 and 2 .

**Recovery of the solid-phase extraction procedure**

To analyse the recovery of xanthones 1 and 2 by SPE, 50 µM of each compound were dissolved in 10 mL of natural sterilized seawater (TSW) and passed through the cartridges, after condition procedure. Chromatographic signals were compared with initial chromatograms, resulting from the injection of the standard solutions before extraction, allowing determining the recovery rate of the extractive process. A recovery rate higher than 95 % was obtained for both compounds (**Figure S1** and **S2**).


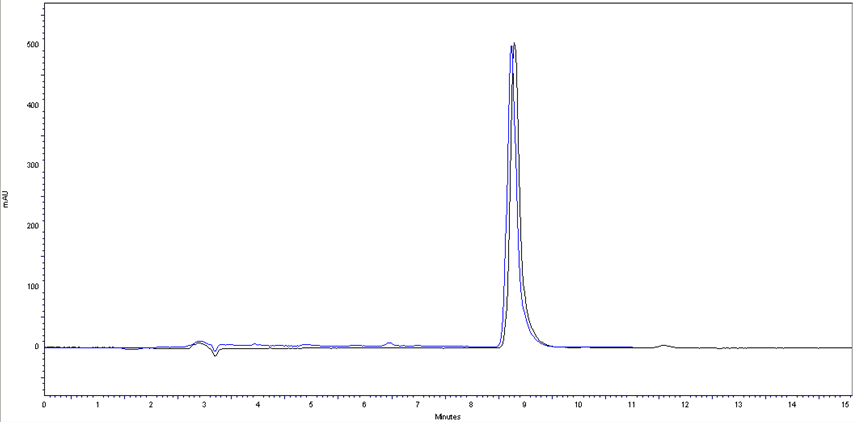


**Figure S1.** Representative chromatograms of Xantifoul **1**. Black: 50 µM of Xantifoul **1** in methanol; Blue: Xantifoul **1** extracted from seawater (pH7.8) by SPE (recovery rate of 101 %).


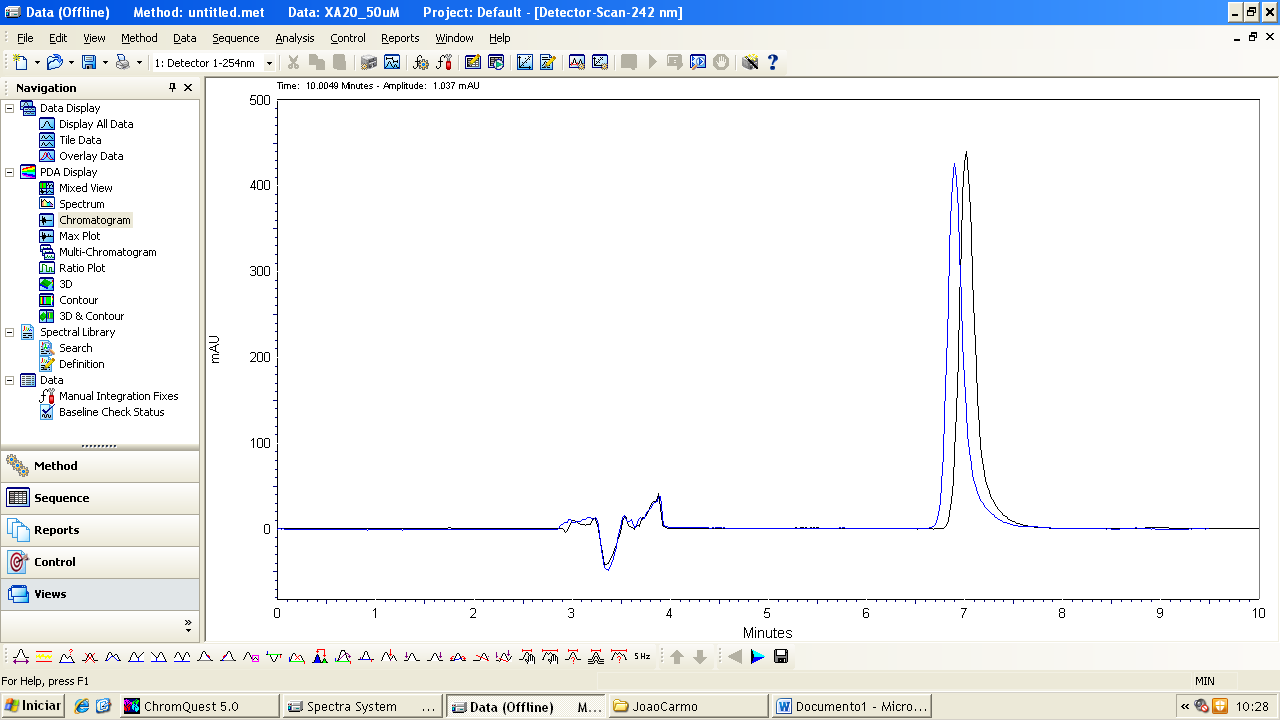


**Figure S2.** Representative chromatograms of Xantifoul **2**. Black: 50 µM of Xantifoul **2** in methanol with 0.1% triethylamine; Blue: Xantifoul **2** extracted from seawater (pH7.8) by SPE (recovery rate of 98 %).

To analyse the recovery of Xantifoul **2** after accelerated degradation assays, three different concentrations (10, 100, and 500 µM) were dissolved in 10 mL of natural sterilized seawater (TSW) and centrifuged. Obtained pellets were dissolved in respective organic solvent and chromatographic signals were compared with initial chromatograms, resulting from the injection of the standard solutions before extraction, allowing determining the recovery rate of the extractive process. A recovery of 74 ± 7 % was obtained for Xantifoul **2** (**Table S3**).

**Table S3.** Accuracy and intra- and inter-day variability (precision) of the newly developed method.

| **Concentration (µM) ^1^** | **Accuracy (% ± SD)** | **Precision (RSD)** |
| --- | --- | --- |
| 10 | 79 ± 2.2 | 6.1 |
| 100 | 66 ± 6.0 | 15.0 |
| 500 | 77 ± 5.4 | 3.0 |

SD: standard deviation; RSD: relative standard deviation; UPW: ultrapure water. ^1^Mean values ± standard deviation of three independent experiences.

**References**

1. ICH, Validation of Analytical Procedures: Text and Methodology. In ***Geneva, Q2(R1), 17 p.***, 2005.
